# Localizing and Tailoring the Debriefing Assessment for Simulation in Healthcare to Optimize Fit

**DOI:** 10.7759/cureus.75570

**Published:** 2024-12-11

**Authors:** Jannet Lee-Jayaram, Kyle Ishikawa, Yu Jin Lee, Len Y Tanaka, Benjamin Lee, Bao Xin Liang, Benjamin W Berg

**Affiliations:** 1 SimTiki Simulation Center, John A. Burns School of Medicine, University of Hawaiʻi at Mānoa, Honolulu, USA; 2 Quantitative Health Sciences, John A. Burns School of Medicine, University of Hawaiʻi at Mānoa, Honolulu, USA; 3 Emergency Medicine, Inha University College of Medicine, Incheon, KOR; 4 Pediatric Critical Care, Kapiʻolani Medical Center for Women and Children, Honolulu, USA; 5 Medicine, John A. Burns School of Medicine, University of Hawaiʻi at Mānoa, Honolulu, USA

**Keywords:** assessment tool, debriefing assessment, healthcare simulation, rater training, simulation in medical education

## Abstract

Introduction

Debriefing in healthcare simulation is helpful in reinforcing learning objectives, closing performance gaps, and improving future practice and patient care. The Debriefing Assessment for Simulation in Healthcare (DASH) is a validated tool. However, localized rater training for the DASH has not been described. We sought to augment DASH anchors with localized notations, localize DASH rater training, assess localized DASH correlation with other debriefing practices/factors, and assess reliability of the localized tool.

Methods

This study was conducted at SimTiki Simulation Center, John A. Burns School of Medicine, University of Hawaiʻi at Mānoa. Three simulation experts without DASH training developed a list of debriefing best practices/factors, reviewed the DASH handbook, and transcribed the DASH Rater Long Form version with example behaviors into a rating document. Research assistants recorded best practices/factor data from archived debriefing videos. Simulation experts independently scored debriefings, resolved discrepancies and added localized criteria to the DASH. Rater calibration was completed with an intraclass correlation (ICC) of 0.884. Raters then independently scored 43 debriefings recorded during July-December 2022. DASH scores were compared to observed debriefing best practices/factors.

Results

The overall DASH behaviors ICC agreement was 0.810 and consistency was 0.825. Behavior scores ranged from 2.45 (SD 0.70) to 4.42 (SD 0.81). The three lowest scoring DASH behaviors were 2A (2.45), 4A (3.41), and 3B (3.44). In behavior 2B regarding realism concerns, there was significant inconsistency in the use of the not applicable (NA) designation. DASH and best practices/factors construct correlation supported convergent validity.

Conclusion

High interrater reliability followed localized rater training and the addition of localized notations to DASH. Correlation with debriefing practices/factors strengthens DASH validity evidence. Lower DASH behavior scores reported generally suggest that the interpretation of DASH scores is best contextualized in a shared localized DASH construct. A comparison of DASH numerical scores between institutions, with different raters, and different cultures may not reflect absolute debriefing quality.

## Introduction

Debriefing promotes lasting learning through facilitator-guided feedback, reflection, analysis, and integration following simulation-based educational events [1]. The purposes of debriefing in simulation in healthcare are to reinforce learning objectives, close performance gaps, and improve future practice and patient care. The facilitator’s/debriefer’s skill in conducting the debriefing influences learning outcomes and therefore should be purposefully planned and structured, based on best-practice and evidence-based guidelines [2]. Tools to assess debriefers and the debriefing process include the Objective Structured Assessment for Debriefing (OSAD), the Debriefing Assessment in Real Time (DART), the Peer Assessment Debriefing Instrument (PADI), and the Debriefing Assessment for Simulation in Healthcare (DASH) [3-6].

The DASH has a long-standing, well-recognized reputation within the healthcare simulation community. It was created with the purpose of yielding reliable data for valid assessments of a debriefer’s competence in a wide variety of healthcare simulation contexts [6]. It is a six-element behaviorally anchored rating scale; each element is further defined by behaviors and observable examples of effective or ineffective behaviors. Elements are rated on a seven-point effectiveness scale ranging from 1, “extremely ineffective/detrimental”, to 7, “extremely effective/outstanding”. The creators intended for the elements to be rated holistically; the behaviors within the elements are not weighted and not intended to be averaged to calculate the element score. The elements are general statements, such as element 2 (“The instructor maintained an engaging context for learning”), while the behaviors are more specific such as behavior 2A (“The instructor clarified the purpose of the debriefing, what was expected of me, and the instructor’s role in the debriefing”). There are six versions of the DASH, both a long and short version scored by the student, external rater/observer, or self-scored by the debriefer (instructor) themselves. Validity evidence for the DASH includes that it was created by simulation experts, after an extensive literature review, through an iterative process, and when used by trained raters, was able to discriminate between varying levels of debriefing performance. Initially described in the setting of video-recorded, standardized, scripted debriefings during DASH rater training, the overall intraclass correlation coefficient (ICC) was 0.74 and Cronbach’s alpha was 0.89 [6]. We chose to use the DASH to rate simulation-based education debriefings at our academic simulation center for purposes of quality assurance, improvement, and faculty needs assessment. Our objectives were to localize and tailor the DASH, localize rater training for the DASH, and assess its reliability and validity while rating actual debriefings.

## Materials and methods

Study setting

This study was conducted at SimTiki Simulation Center, John A. Burns School of Medicine, University of Hawaiʻi at Mānoa. At our academic simulation center based within a medical school, the learner population includes medical students, residents, practicing healthcare providers, faculty, advanced practice providers, paraprofessionals, and community educators. Simulation course directors are faculty who utilize the center’s resources and coordinate all aspects of simulation courses, including facilitator/debriefer recruitment and training. As part of our simulation center’s quality assurance and improvement processes, debriefings are routinely audio-video-recorded from fixed ceiling-mounted cameras and microphones. Videos are securely stored according to center policies and debriefers are annually offered the opportunity to review their debriefings while completing the DASH Instructor Version (IV). The simulation center faculty review completed DASH forms and video recordings of debriefings only at the request of each individual facilitator, to support their professional development. In 2023, the simulation center faculty conducted a quality improvement review of archived video-recorded debriefings to assess debriefing quality, monitor debriefing practices, and determine faculty development needs. The DASH Rater Version (RV) Long Form was selected for the detail of the debriefing quality afforded by scoring each behavior within each element and precision in identifying faculty development needs.

Video-recorded debriefings were chosen from a convenience sample of all debriefings conducted from July to December of 2022, during the first half of the academic school year. The videos were selected by the simulation center’s operations director who routinely records debriefings as part of the simulation center’s annual effort to provide debriefers opportunities to review their practice. The operations director was not involved in this study. Excluded were debriefings during this interval that were not video-recorded, summative assessment sessions, videos with insufficient video or audio quality, debriefings from faculty development programs, or incomplete debriefing videos. The video review included only debriefings and did not include orientation or simulation scenario content. Debriefings that immediately followed simulation scenarios were utilized. Debriefings were conducted by medical school faculty with variable experience and training in simulation-based education and for student learners at various levels including medical students and medical residents.

This study was determined not to be a human subjects research by the University of Hawai‘i Human Studies Program under the Office of Research Compliance (protocol number 2022-00495).

Design

Two medical student research assistants who did not rate video debriefings reviewed the video-recorded debriefings. A checklist of debriefing practices/factors that the simulation center faculty determined to comprise observable elements of high-quality debriefings was completed by the research assistants as they observed the recordings [1,2,7,8]. Debriefing practices/factors included the following items: whether a debriefing framework was used, if the scenario content was matched to the clinical or other expertise of the debriefer, the number of students in the debriefing, the number of debriefers, presence of a clear transition from scenario to debriefing, if the debriefer appeared to be using notes or a written guide, if the learners/debriefer were seated, if a white board was used by learners/debriefer, if there was written material shared with the learners, if the debriefer used an assessment tool during the debriefing, if the learners/debriefer made a statement about quality of the performance, if the scenario video review was used during the debriefing, duration of debriefing, number of talking points, and if any event during the debriefing impacted it. Answers on the checklist for dichotomous debriefing practices/factors included “yes”, “no”, and “can’t tell”. Numerical answers were entered for quantitative debriefing practices/factors. Each video was reviewed by one research assistant.

The three DASH raters each had more than 15 years of healthcare simulation experience, were simulation center faculty, Certified Healthcare Simulation Educator (CHSE) certified, and were simulation education researchers. None of the raters had previous formal DASH rater training. None of the rated debriefings included any of the three raters as debriefers. Some rated debriefings were conducted in courses that the raters routinely facilitate and debrief.

Rater training and calibration 

Rater training used a frame of reference (FOR) approach [9-11]. Raters first reviewed the DASH rater’s handbook. The handbook contains detailed descriptions of all six elements and their behaviors, with specific examples of effective and ineffective behaviors. The DASH was then transcribed into an editable rating tool document including examples of the effective and ineffective behaviors from the handbook listed beneath the description of each element’s behaviors. Each rater independently scored a debriefing from a set of training video debriefing recordings. Following this, they met on a virtual platform and discussed scores for each behavior. Discrepancies were further explored and resolved, with localized notations augmenting the native DASH behaviors in the editable document to guide raters towards an agreed upon appropriate score. The raters added localized notations to further guide the numerical choice on the native DASH anchors on the seven-point effectiveness rating scale. For example, a localized notation to the descriptor under the rating of 1 was “debriefer is a monster” as the raters came to the agreement that such phrasing would allow them to understand when to score a behavior as a 1. Additionally, the raters added notations to guide appropriate scoring for each behavior within each element. An example of a localized notation was “downgrade one if there are moments where student was made to feel bad” in Behavior 2E (Participants could share thoughts and emotions without fear of being shamed or humiliated). Discussion about each behavior clarified which should always be rated and which could potentially be not applicable (NA), and the raters added this notation on the editable DASH document to read “always score this”. This process was iteratively repeated a total of four times, with additional localized notations placed in the editable DASH document after each round. Twelve separate notations were added in round 1, 17 added in round 2, 11 in round 3, and 3 in round 4. After the fourth round, the raters felt that saturation of localized notations within the document was achieved, and that the raters had a shared understanding of how to use the DASH within the local educational context.

In the final step of rater training, the three raters independently scored five videos that were in a unique randomized sequence for each rater to minimize any learning effect on the scores. These videos were not included in the main analysis and only used for the purpose of calibration. Individual items of the DASH had a Cronbach’s alpha of 0.961 for internal consistency. The overall scores demonstrated an ICC agreement of 0.884 and consistency of 0.881 among raters. At that time, rater calibration was determined to be sufficient.

The raters then went on to view and score 50 debriefings independently. The debriefings were uniquely randomized for each rater again to minimize any learning effect on their scores.

Element 1 behaviors were not scored for any of the videos as pre-briefing was not recorded and therefore could not be evaluated. During rater calibration, three behaviors were determined as eligible for an NA designation: behavior 2B addressing concerns about realism, behavior 4D addressing the use of recordings to support analysis, and behavior 4E addressing the upset participant. Behaviors that received an NA designation from any of the raters were not included in the final numerical analysis.

Statistical analysis

The ICC agreement and consistency among raters were calculated for 16 DASH behaviors. For statistical calculation, the unit of measurement was the average rater score for each debriefing. Means and standard deviations were presented for each DASH behavior, alongside ICC scores. The association between specific components of the DASH and debriefing practices/factors were tested using the Wilcoxon rank sum test for factors with two levels and the Kruskal-Wallis rank sum test for factors with more than two levels. Since these tests were non-parametric, the medians and interquartile values (quartiles 1 and 2) were presented for each debriefing practice/factor.

Software Packages

Cronbach’s alpha was calculated using the *ltm *package [12] and ICC was calculated using the *irr *package [13]. Boxplots were created using the *ggpubr *package [14]. All calculations were performed in R version 4.3.2 [15].

## Results

Of 50 video-recorded debriefings, seven were excluded from final evaluation as five were case discussions without simulation and two were incomplete recordings. A total of 49% of the rated debriefings were conducted in courses that at least one of the raters routinely facilitate and debrief. Video recordings captured 25 unique debriefers. See Table 1 for demographic characteristics of debriefers. There was an even gender distribution among rated debriefers, with an average age of 50 years, indicating physician faculty who were mid-career. Half the debriefers completed the simulation-based healthcare education training offered quarterly at the simulation center. While the average length of time debriefers had been teaching with simulation at the center was 16 years, the number of debriefings within the past year averaged only 2.68.

**Table 1:** Debriefer demographic characteristics DASH, Debriefing Assessment for Simulation in Healthcare

Characteristic	
Gender, n (%)	
Female	11 (46%)
Male	13 (54%)
Unknown	1
Age, mean ± SD (years)	50 ± 12
Unknown	1
Completed general simulation training, n (%)	14 (56%)
Total debriefing experience, mean ± SD (years)	16 ± 18
Number of debriefings conducted in the last year, mean ± SD	2.68 ± 2.44
Completed DASH self-evaluation in the past, n (%)	6 (24%)

The overall DASH behaviors ICC agreement between raters was 0.810 and consistency was 0.825. Individual behavior agreement and consistency, with individual behavior scores are given in Table 2.

**Table 2:** Individual DASH behavior scores with ICC among raters DASH, Debriefing Assessment for Simulation in Healthcare; ICC, intraclass correlation

DASH	Mean (SD)	ICC agreement	ICC consistency
Element/behavior			
2. Maintains an engaging learning environment			
A. Debriefer clarified purpose of debriefing, what was expected of participants, and the debriefer’s role in the debriefing	2.45 (0.70)	0.641	0.690
C. Debriefer conveyed respect for participants	4.15 (0.74)	0.720	0.745
D. The focus was on learning and not making people feel bad about making mistakes	4.39 (0.63)	0.723	0.720
E. Participants could share thoughts and emotions without fear of being shamed or humiliated	4.24 (0.65)	0.733	0.741
3. Structures the debriefing in an organized way			
A. The conversation progressed logically rather than jumping around from point to point	3.65 (0.96)	0.813	0.832
B. Near the beginning of the debriefing, participants were encouraged to share their genuine reactions to the case and the debriefer seemed to take the remarks seriously	3.44 (1.24)	0.883	0.884
C. In the middle, the debriefer helped participants analyze actions and thought processes as they reviewed the case	3.69 (0.92)	0.765	0.836
D. At the end of the debriefing, there was a summary phase where the debriefer helped tie observations together and related the case to way(s) participants could improve their future clinical practice	3.50 (1.05)	0.817	0.864
4. Provokes engaging discussion			
A. The debriefer used concrete examples (not just abstract or generalized comments) to get participants to think about their performance	3.41 (0.92)	0.754	0.783
B. The debriefer’s point of view was clear; participants didn’t have to guess what the debriefer was thinking	3.95 (0.68)	0.584	0.580
C. The debriefer listened and made people feel heard by trying to include everyone, paraphrasing, and using non-verbal actions like eye contact and nodding, etc.	4.23 (0.85)	0.752	0.806
5. Identifies and explores performance gaps			
A. Participants received concrete feedback on their individual performance or that of their team based on the debriefer’s honest and accurate view	3.71 (0.88)	0.722	0.733
B. The debriefer helped explore what participants were thinking or trying to accomplish at key moments	3.52 (0.90)	0.739	0.782
6. Helps trainees achieve or sustain good future performance			
A. The debriefer helped participants learn how to improve weak areas or how to repeat good performance	3.57 (0.91)	0.719	0.778
B. The debriefer was knowledgeable and used the knowledge to help participants see how to perform well in the future	4.42 (0.81)	0.729	0.736
C. The debriefer made sure the discussion covered important topics	4.25 (0.94)	0.749	0.791

Scores for DASH behaviors ranged from 2.45 (SD 0.70) to 4.42 (SD 0.81). The three lowest scoring DASH behaviors were behavior 2A (“The instructor clarified the purpose of the debriefing, what was expected of participants, and the debriefer’s role in the debriefing”) with a mean score of 2.45, behavior 4A (“The debriefer used concrete examples, not just abstract or generalized comments, to get participants to think about their performance”) with a mean score of 3.41, and behavior 3B (“Near the beginning of the debriefing, participants were encouraged to share their genuine reactions to the case and the debriefer seemed to take the remarks seriously”) with a mean score of 3.44.

**Figure 1:**
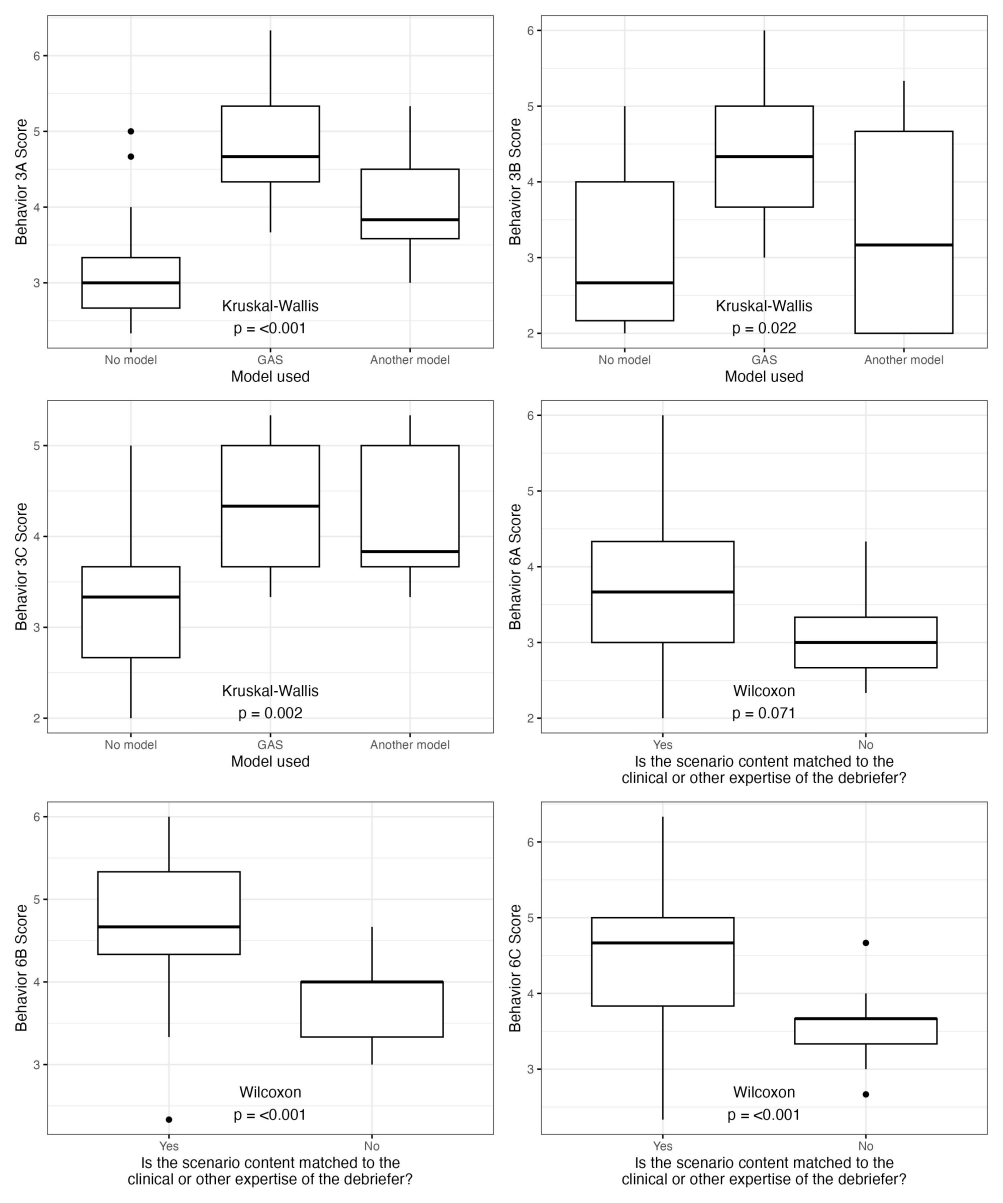
DASH behavior associations with observed debriefing practices/factors DASH, Debriefing Assessment for Simulation in Healthcare; GAS, gather-analyze-summarize

Debriefing practices/factors identified on the video review that were also measured by the DASH correlated with higher median DASH scores in those behaviors, as seen in Figure 1. Higher DASH scores in behavior 3A (“The conversation progressed logically rather than jumping around from point to point”), behavior 3B (“Near the beginning of the debriefing, participants were encouraged to share their genuine reactions to the case and the debriefer seemed to take the remarks seriously”), and behavior 3C (“In the middle, the debriefer helped participants analyze actions and thought processes as they reviewed the case”) were associated with the debriefer using a gather-analyze-summarize (GAS) model or another model compared to no model. These behaviors are under overall element 3 (“Structures the debriefing in an organized way”). Higher DASH scores in behavior 6A (“The debriefer helped participants learn how to improve weak areas or how to repeat good performance”), behavior 6B (“The debriefer was knowledgeable and used that knowledge to help participants see how to perform well in the future”), and behavior 6C (“The debriefer made sure the discussion covered important topics”) were associated with the debriefing practice/factor which identified that the scenario content was matched to the clinical or other expertise of the debriefer. These behaviors are under overall element 6 (“Helps trainees achieve or sustain good future performance”).

In element 4 (“Provokes engaging discussion”), the two behaviors that scored as NA 99% of the time were D (“The debriefer used video or recorded data to support analysis and learning) and E (“If someone got upset during the debriefing, the debriefer was respectful and constructive in trying to help them deal with it”). For 39.5% of the debriefings, the raters could not agree on the use of the NA designation for behavior 2B (“The debriefer acknowledged concerns about realism and helped participants learn even though the cases were simulated”).

## Discussion

We report that higher DASH behavior scores correlated with debriefing practices/factors purported to be measured by DASH, supporting construct/convergent validity evidence. The correlation of debriefing practices/factors that we identified by independent non-DASH guided observation of debriefing strengthens validity evidence for DASH. Higher scores were noted for behaviors in element 3 that measures if the debriefing was structured in an organized way, correlating with the observation that a debriefing framework was employed during the debriefing. Additionally, higher scores were noted when the scenario content was matched to the debriefer’s expertise. Behavior 6B measures if the debriefer was knowledgeable and used that knowledge to help participants see how to perform well in the future and correlated with the observation that the debriefer subject matter expertise matched the simulation content.

Debriefing practices/factors identified by the video review that incorporated and correlated with concepts of DASH behaviors, as noted in Figure 1, provide evidence of convergent validity. This extends findings from student DASH scores in other studies, which correlated to debriefing practices/factors including improved simulation design, a psychologically safe environment, decreased learner stress and a smaller number of students [16].

Interrater reliability (IRR) was high using a localized rater training process, including the addition of the positive and negative effective behavior lists noted in the DASH rater training handbook and localized notations to the DASH rating form. Reliability and in part validity of the rating may depend on who is scoring the DASH. High interrater reliability has been reported by experts scoring debriefings using the tool. However, when non-simulationist video raters who received minimal debriefing training used the DASH to score video-recorded debriefings in a resource-poor area, their IRR scores were poor [17]. This highlights the improved utility and quality that expert raters confer on DASH scores, and may have implications for the interpretation of scores. However, despite the use of trained simulation expert raters, incorporation of our localized notations failed for behavior 2B (“The debriefer acknowledged concerns about realism and helped participants learn even though the cases were simulated”). Although the localized notation read that this element would be marked NA unless the students brought up concerns about fidelity or real-life comparisons, raters did not agree on the use of the NA designation in nearly 40% of the debriefings. Descriptions about challenges or inconsistencies with DASH NA designations have not been described, yet it could be anticipated that this behavior may be more inconsistently designated NA as it requires rater interpretation of discussion topics rather than simply noting the use of video for debriefing.

DASH ratings of the same debriefing by students, debriefers themselves, and expert raters have demonstrated significant differences, with expert rater scores being lower and student scores higher [18]. In fact, the majority of literature reports DASH scores by learners using the DASH Student Version (SV), although the validated DASH-RV tool was developed in the context of trained raters with education or simulation backgrounds. Interrater reliability calculations have not been reported when the DASH has been completed by learners only. Understanding subjective learner debriefing experiences and perceptions is valuable; however, the perspective of an expert debriefer, knowledgeable in debriefing methods, is likely more valuable and effective for faculty/instructor feedback and development. Many studies report high DASH-SV ratings by students with element scores in the high 5-6 range, with a score of 5 represented on the scale as mostly effective/good and a score of 6 being consistently effective/good [18-20]. One study of interprofessional education (IPE) for emergency department orientation reported very high novice nurses' DASH-SV ratings of nurse and physician educators, with a mean overall DASH score of 6.97 [21]. Another study using the DASH-SV rated novice instructors before and after a debriefing course, and explicitly excluded participants with any previous debriefing experience [22]. Pre-training, learners rated these novices with no debriefing experience median DASH scores of 4 and higher, further demonstrating the tendency for student DASH scores to be inflated. After the debriefing course, the novice debriefers’ student DASH scores were higher, indicating that learners can perceive changes in debriefing quality. When students rated debriefings with telepresent debriefers compared to in-person debriefers, absolute DASH scores were in the high range, however with significantly higher scores for the in-person group [23-25]. For student DASH scores, the anchor beneath the rating scale cannot likely be applied accurately, and rather, trends or comparisons for student DASH scores may be more meaningful. The interpretation of student DASH scores in those studies as evidence of effective debriefing may not be as valid as the conclusion that students prefer in-person debriefings compared to telepresent debriefings or that training improved novice debriefer skills. Comparing student DASH scores between faculty and peers to determine that both are acceptable debriefers for the same cohort of students could also be a valid conclusion [26,27].

DASH may not provide a one-size-fits-all score that can be interpreted and compared across all circumstances and educational environments. Its meaning and use may be valuable within a specific setting, to use as comparisons between debriefings, debriefers, debriefing methods, over time or reflective of other interventions. Our DASH behavior scores were lower than those previously reported. This could mean that the quality of our debriefings is significantly lower, our raters were prone to severity bias, or that the meaning of quality is variable depending on the local educational culture. In one study of senior nursing students providing peer debriefing, average DASH-RV element scores were >5 [27]. These were ratings by nurse alumni familiar with the curriculum with an IRR of 0.751. However, in another study evaluating the impact of a cognitive debriefing aid on new simulation fellows' debriefing, mean element scores ranged from 4.5 to 4.9 [28], closer in range to the reported scores from the original DASH report [6]. DASH developers had raters score standardized, scripted debriefings that represented poor, average, and superior debriefings. The mean scores for these three levels of performance were 2.18, 4.77, and 5.35, respectively, indicating that average debriefing scores would be expected in the range of 4.

While the goal is to develop debriefers with superior skills, the majority of faculty who debrief at our center are not simulationists. In this cohort, though we had mid-career faculty who had been teaching with simulation for over a decade, only half had completed our regularly offered faculty development course on simulation-based healthcare education. Additionally, the frequency of debriefing was not high, as individual facilitators conducted an average of two to three debriefings in the last year. At our center, apart from the simulation center faculty, facilitators conduct at most six post-simulation debriefings per year and some only once yearly. We would not expect DASH behavior scores consistently in the 6 and above range. Through our rater training process, raters came to a shared understanding of what was expected when rating each behavior and we localized what was expected of an average debriefing. The interpretation of DASH scores should be localized and viewed in the context of a shared local meaning of ratings, elements, and behaviors. A comparison of DASH numerical scores between institutions, with different raters, different institutional and national cultures cannot provide information about absolute quality of debriefing, only about the localized assessment of the debriefings. Our lower scores likely represent localized expectations rather than universally applicable quality.

Another challenge when interpreting and generalizing conclusions based on absolute DASH scores is the variability in reporting of scores. Studies variably report DASH scores as means of each element, or as an overall mean on the seven-point effectiveness scale, or as a total sum of the scores of each element. DASH mean scores have been reported in the 150s, 20s, and single digits [18,20,27]. Reported scores also vary because of the exclusion or inclusion of element 1, which occurs at the start of the educational activity, not during the debriefing.

Practical recommendations

Rather than using one-time DASH scores, especially student-scored, as general evidence of debriefing effectiveness, the applicability and utility of DASH should be considered through a more targeted and purposeful lens. Suggested DASH applications include faculty development, linking DASH scores to other outcomes, assessment of program quality, providing feedback, and as a requirement for certification [29,30]. We propose that localized rater training and calibration is required when using the DASH for decision-making or assessing quality and value of debriefings. This would ensure that there is proper understanding, interpretation, and utilizatization of the tool within the unique educational culture of each institution. Requirements to establish localized training may include a repository of recordings of real debriefings from debriefers of varying skills, faculty with expertise in simulation-based healthcare education, and time set aside for iterative refinements to the localized tool. Refinements should focus on achieving agreement on completion of rated items, a common understanding of the rating scale values, and incorporating specific descriptive localized notations for behaviors. As a tool for identifying areas for quality improvement and faculty development, attention can be focused on the lowest scoring behaviors within the institution, without necessary regard for the absolute number.

Limitations

The study was not without limitations. Video recordings did not include pre-briefings; therefore, DASH element 1 could not be scored. The quality of the pre-briefing, especially in preparing learners to participate in the debriefing, could impact the quality of the debriefing and may provide useful context for interpreting other DASH behavior scores.

Behaviors 2B, 4D, and 4E were eligible for NA designations as scores depended on the presence of a learner behavior or use of video-recorded data during debriefing. While raters achieved high agreement on the NA designation for behaviors regarding the upset learner or use of video-recorded data, there was poor agreement regarding learner concerns about realism. The upset participant is an unusual occurrence and video-debriefing is not routinely used in our center; therefore, additional localized notations beyond the eligibility of the NA designation was not discussed. However, focused attention during rater calibration may be required to identify when a learner has difficulty engaging in the fiction contract, as well as evaluating how the debriefer addresses the issue.

The video review did not include events that occurred during simulation, so raters could not assess if debriefings sufficiently covered and explored all significant events. Objectives of the sessions were not provided to reviewers. However, we assert that by the end of a high-quality debriefing, an observer should be clear about the objectives of the simulation activity and if student gaps were identified and explored. Additionally, since nearly half of the rated debriefings were conducted in courses that at least one of the raters routinely facilitate and debrief, the raters had some insight to inform rating of debriefings.

The traditional FOR rater training could not be undertaken as all our recorded debriefings were actual educational events, not scripted, and were not previously ascribed DASH scores. However, now we have 43 debriefing recordings that have been scored by expert simulationists, and from this stock we could choose debriefings representative of poor, average, and superior quality and utilize those for FOR training in our institution.

Finally, the reliability of the ICC results may be limited due to the relatively small sample size of the 43 videos included in the review.

## Conclusions

The DASH does not provide a one-size-fits-all score that can be interpreted in all circumstances and educational environments. It is imperative to establish DASH reliability in the local context. We plan to utilize the localized DASH-SV similar to this study to explore student assessment of debriefing and the impact of a locally configured tool. Those results will be used to explore what aspects of debriefing our students value by assessing relative scoring of various elements/behaviors and how those compare with expert-rated scores from this study, especially when the same debriefers are assessed. The localized DASH-SV will also provide continued surveillance of quality assurance, assess faculty development needs and provide actionable feedback for faculty development.
